# Piperidine-4-Carboxamides Target DNA Gyrase in Mycobacterium abscessus

**DOI:** 10.1128/AAC.00676-21

**Published:** 2021-07-16

**Authors:** Dereje Abate Negatu, Andreas Beuchel, Abdeldjalil Madani, Nadine Alvarez, Chao Chen, Wassihun Wedajo Aragaw, Matthew D. Zimmerman, Benoît Laleu, Martin Gengenbacher, Véronique Dartois, Peter Imming, Thomas Dick

**Affiliations:** a Center for Discovery and Innovation, Hackensack Meridian Health, Nutley, New Jersey, USA; b Center for Innovative Drug Development and Therapeutic Trials for Africa (CDT-Africa), Addis Ababa University, Addis Ababa, Ethiopia; c Institut für Pharmazie, Martin-Luther-Universität Halle-Wittenberg, Halle, Germany; d Medicines for Malaria Venture, Geneva, Switzerland; e Department of Medical Sciences, Hackensack Meridian School of Medicine, Nutley, New Jersey, USA; f Department of Microbiology and Immunology, Georgetown University, Washington, DC, USA

**Keywords:** *Mycobacterium abscessus*, NTM, nontuberculous mycobacteria, MMV688844, DNA gyrase

## Abstract

New, more-effective drugs for the treatment of lung disease caused by nontuberculous mycobacteria (NTM) are needed. Among NTM opportunistic pathogens, Mycobacterium abscessus is the most difficult to cure and intrinsically multidrug resistant. In a whole-cell screen of a compound collection active against Mycobacterium tuberculosis, we previously identified the piperidine-4-carboxamide (P4C) MMV688844 (844) as a hit against M. abscessus. Here, we identified a more potent analog of 844 and showed that both the parent and improved analog retain activity against strains representing all three subspecies of the M. abscessus complex. Furthermore, P4Cs showed bactericidal and antibiofilm activity. Spontaneous resistance against the P4Cs emerged at a frequency of 10^−8^/CFU and mapped to *gyrA* and *gyrB* encoding the subunits of DNA gyrase. Biochemical studies with recombinant M. abscessus DNA gyrase showed that P4Cs inhibit the wild-type enzyme but not the P4C-resistant mutant. P4C-resistant strains showed limited cross-resistance to the fluoroquinolone moxifloxacin, which is in clinical use for the treatment of macrolide-resistant M. abscessus disease, and no cross-resistance to the benzimidazole SPR719, a novel DNA gyrase inhibitor in clinical development for the treatment of mycobacterial diseases. Analyses of P4Cs in *recA* promoter-based DNA damage reporter strains showed induction of *recA* promoter activity in the wild type but not in the P4C-resistant mutant background. This indicates that P4Cs, similar to fluoroquinolones, cause DNA gyrase-mediated DNA damage. Together, our results show that P4Cs present a novel class of mycobacterial DNA gyrase inhibitors with attractive antimicrobial activities against the M. abscessus complex.

## INTRODUCTION

While the incidence of tuberculosis has declined, infections by nontuberculous mycobacteria (NTM) are increasing (1–3). NTM lung disease is the most common clinical presentation and is primarily caused by members of the Mycobacterium abscessus and Mycobacterium avium complexes (4). Although they are close relatives of Mycobacterium tuberculosis, these NTM species exhibit differential pathogenesis due to expression of novel surface lipids, adaptation to both host and environmental niches, and acquisition of novel virulence factors (5). In addition, NTM demonstrate intrinsic resistance to a broad range of antibiotics (3, 6). The current treatments for NTM lung disease vary by species and differ from the standard four-drug TB chemotherapy (7, 8). M. abscessus next to M. avium presents the most difficult to cure NTM disease (9, 10). Its treatment regimen typically consists of a macrolide (azithromycin or clarithromycin) combined with parenteral drugs (amikacin, imipenem-cefoxitin, tigecycline) and may include other oral antibiotics (clofazimine, linezolid) for a minimum of 12 months (8). However, with cure rates of less than 50%, treatment outcomes for patients with M. abscessus infections are unsatisfactory (11, 12). Contributing to the poor treatment outcomes may by the ability of M. abscessus to grow as biofilms in patients (13, 14). Most clinically used anti-M. abscessus drugs are bacteriostatic and do not show antibiofilm activity, which may contribute to the ineffectiveness of current regimens (15). Thus, new, more-efficacious drugs are needed to curb the rise of NTM infections, including “incurable” M. abscessus lung disease (16–18).

Over the past 2 decades, a large number of novel hits active against M. tuberculosis were identified through successful whole-cell screening campaigns, leading to a reenergized tuberculosis drug discovery and development pipeline (https://www.newtbdrugs.org/). We and others have shown that focused libraries of compounds active against M. tuberculosis provide an attractive source of hits active against NTM (17–21). In one such screen, using the Pathogen Box collection from Medicines for Malaria Venture (https://www.mmv.org/mmv-open/pathogen-box), we identified the piperidine-4-carboxamide (P4C) MMV688844 (844) as a hit active against our M. abscessus screening strain M. abscessus subsp. *abscessus* Bamboo (21). This compound was originally identified as the noncytotoxic hit TCMDC-143649 in GlaxoSmithKline’s whole-cell screen against M. tuberculosis (22). Based on *in silico* analyses, it was proposed that 844 targets mycobacterial ABC transporters (23).

Here, we show that 844 and a more potent analog are broadly active against a collection of strains representing the three subspecies of the M. abscessus complex. We demonstrate that the P4Cs show attractive bactericidal and antibiofilm activity and determine their mechanism of action. Genetic, biochemical, and reporter strain analyses indicate that P4Cs target mycobacterial DNA gyrase, rather than ABC transporters as proposed earlier (23). Thus, this work identified a novel mycobacterial DNA gyrase inhibitor.

## RESULTS AND DISCUSSION

### 844 and improved analog 844-TFM are active against M. abscessus subspecies.

**FIG 1:**
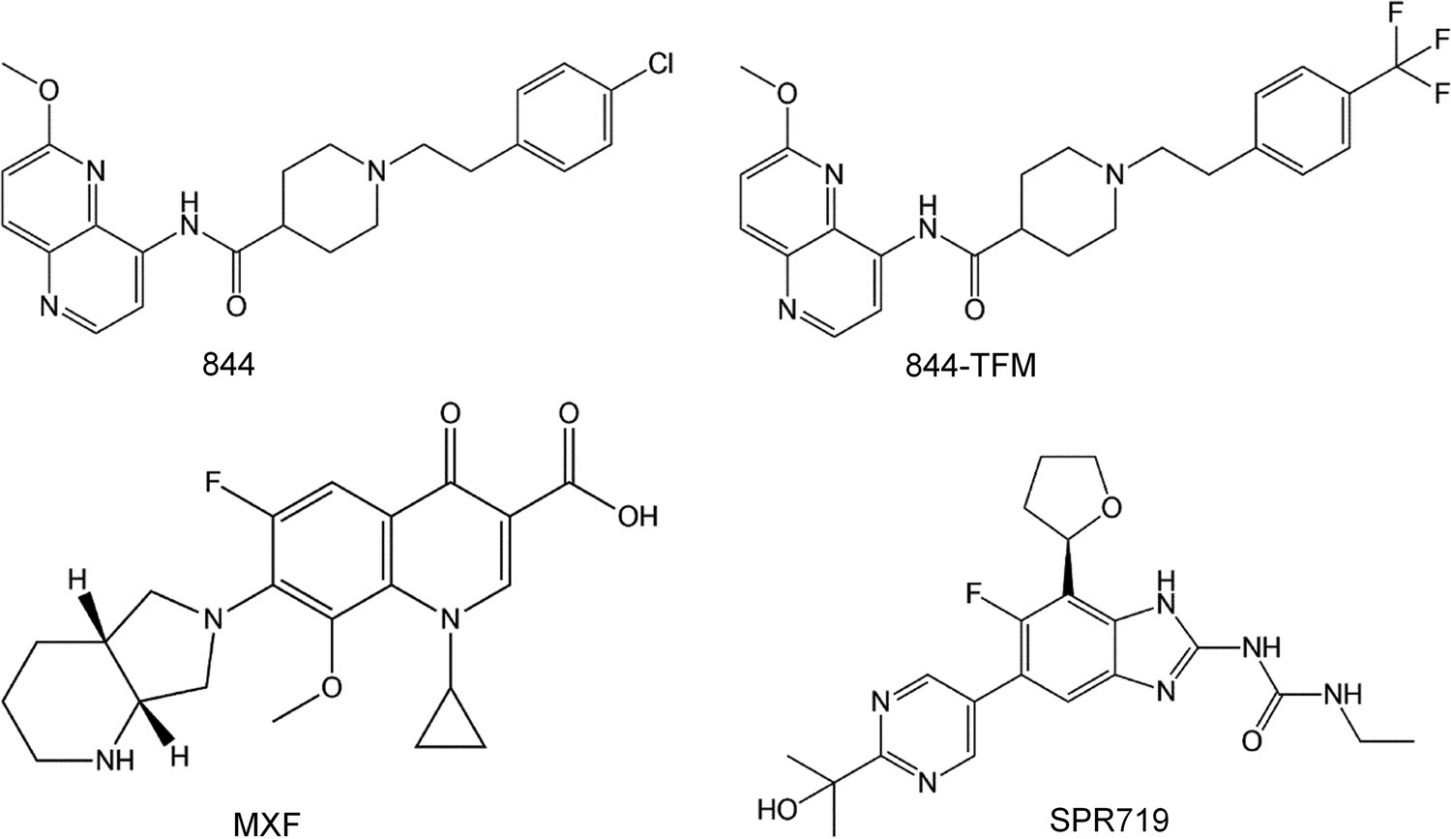
Structures of piperidine-4-carboxamides 844 and 844-TFM, and the DNA gyrase inhibitors moxifloxacin (MXF) and SPR719.

We previously reported the activity of 844 (Fig. 1) against the clinical isolate M. abscessus subsp. *abscessus* Bamboo (21). To determine the potential usefulness of this new whole cell active for the treatment of M. abscessus disease, we measured its activity against reference strains representing the three subspecies of the M. abscessus complex as follows: M. abscessus subsp. *abscessus* ATCC 19977, M. abscessus subsp. *bolletii* CCUG 50184T, M. abscessus subsp. *massiliense* CCUG 48898T, and a collection of clinical isolates. 844 retained activity against the M. abscessus complex strains with MICs ranging from 6 to 14 μM (Table 1).

**TABLE 1:** Growth inhibitory activity of 844 and 844-TFM against subspecies reference strains and clinical isolates of the M. abscessus complex

M. abscessus subspecies^*a*^	*erm*(41) sequevar^*b*^	CLR susceptibility^*b*^	MIC^*c*^ (μM)				
			844	844-TFM	MXF	SPR719	CLR
Reference strains							
* abscessus* ATCC 19977	T28	Resistant	8.0	1.5	3	0.6	2.0
* bolletii* CCUG 50184T	T28	Resistant	14.0	2.0	6.3	1.5	1.2
* massiliense* CCUG 48898T	Deletion	Sensitive	14.0	2.0	6.3	3.0	0.2
Clinical isolates							
* abscessus* Bamboo	C28	Sensitive	12.0	1.5	4.0	0.8	0.4
* abscessus* K21	C28	Sensitive	14.0	3.5	3.0	1.5	0.6
* abscessus* M9	T28	Resistant	6.3	3.1	1.5	1.5	3.2
* abscessus* M199	T28	Resistant	6.3	6.3	2.5	1.5	3.2
* abscessus* M337	T28	Resistant	6.3	3.1	1.5	0.8	3.2
* abscessus* M404	C28	Sensitive	6.3	1.6	3.0	1.5	0.8
* abscessus* M421	T28	Resistant	6.3	1.6	1.5	0.8	3.2
* bolletii* M232	T28	Resistant	6.3	1.6	1.5	1.5	3.2
* bolletii* M506	C28	Sensitive	6.3	3.1	2.5	1.5	0.8
* massiliense* M111	Deletion	Sensitive	12.5	6.3	3.2	6.3	0.2

a M. abscessus Bamboo (42), K21 (43), and the M strains (44) have been described previously.

b *erm*(41), ribosome methylase gene responsible for inducible clarithromycin (CLR) resistance. C28 and deletion sequevars of the gene are susceptible to clarithromycin, while the T28 sequevar confers inducible resistance against CLR (55).

c The DNA gyrase inhibitors moxifloxacin (MXF) and SPR719 are included for comparison and the ribosome inhibitor CLR as assay control. MIC determinations were carried out three times independently in technical duplicates, and mean values are shown.

Next, we generated a set of analogs, demonstrating a dynamic structure activity relationship (A. Beuchel, D. Robaa, D. A. Negatu, A. Madani, N. Alvarez, M. D. Zimmerman, A. Richter, L. Mann, S. Hoenke, R. Csuk, T. Dick, and P. Imming, unpublished data). By substituting the chlorine in position 4 of the phenyl moiety of 844 with a trifluoromethyl group (Fig. 1), we identified an ∼8-fold more potent analog (844-TFM). Similar to 844, 844-TFM retained activity across the M. abscessus complex with MICs ranging from 1.5 to 6 μM (Table 1).

### P4Cs are bactericidal and active against M. abscessus biofilm cultures.

**FIG 2:**
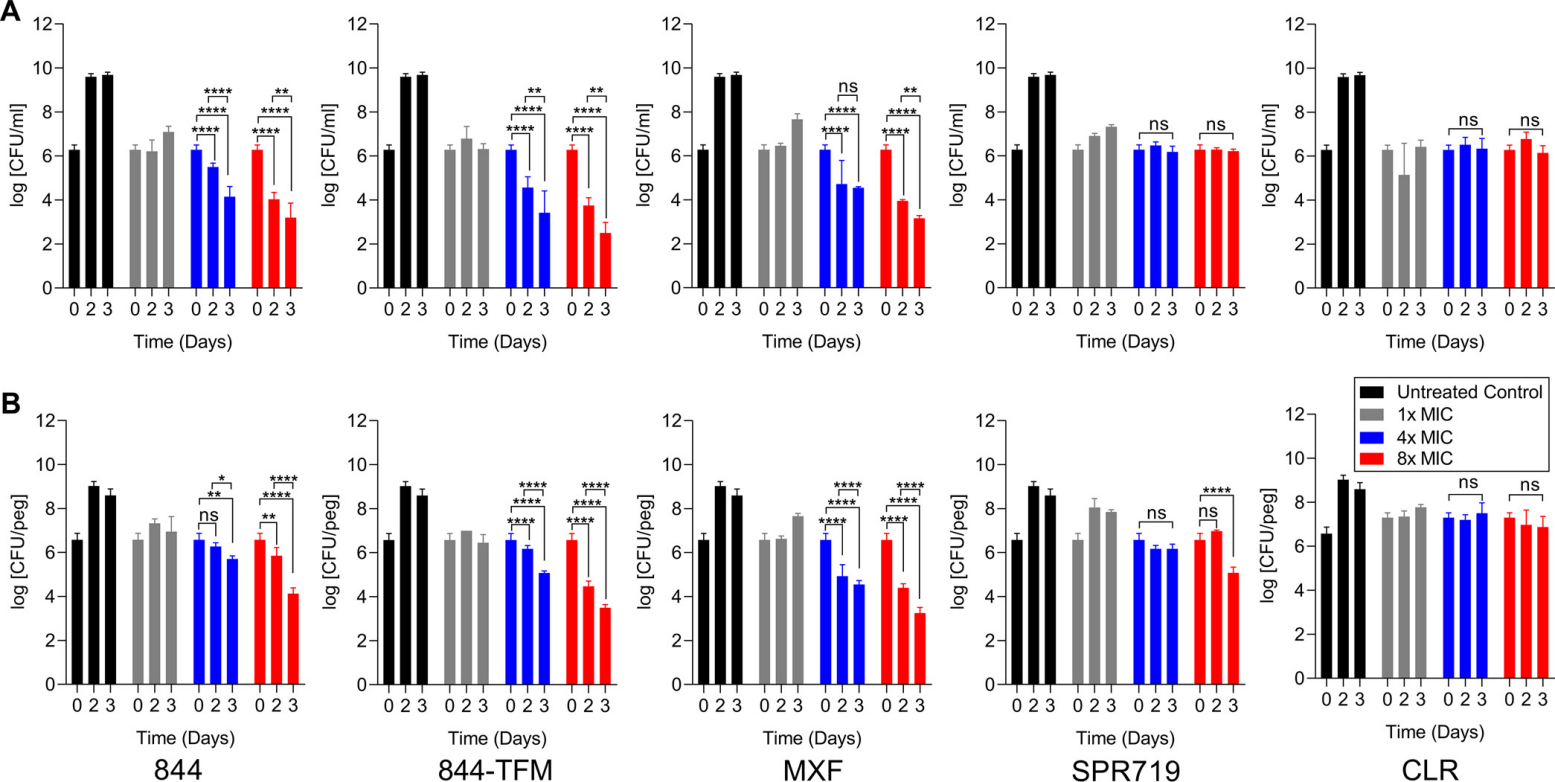
Bactericidal and antibiofilm activity of 844 and 844-TFM against M. abscessus. (A) Exponentially growing suspension cultures of M. abscessus subsp. *abscessus* ATCC 19977 were treated with 1×, 4×, and 8× MIC of 844 or 844-TFM, moxifloxacin (MXF), SPR719, or clarithromycin (CLR), and CFU were enumerated by plating samples on agar after 2 and 3 days. (B) Exponentially growing biofilm cultures were treated with 1×, 4×, and 8× MIC of 844, 844-TFM, MXF, SPR719, or CLR, and surface-attached CFU were enumerated by suspending bacteria and plating on agar after 2 and 3 days. MXF, SPR719, and CLR are included for comparison (MXF, SPR719) or as control (CLR). Experiments in panels A and B were carried out three times independently, and the results are represented as mean values with the standard deviations displayed as error bars. A two-way ANOVA multiple comparison test was performed using GraphPad Prism 8 software to compare treated groups with untreated day 0 CFU.

We previously reported reduced viability of M. abscessus subsp. *abscessus* Bamboo treated with 844 in broth culture, suggesting bactericidal activity of the compound (21). To confirm and characterize the bactericidal activity of the class, we determined time-concentration kill for 844 and 844-TFM against the reference strain M. abscessus subsp. *abscessus* ATCC 19977. Both compounds were bactericidal in planktonic cultures (Fig. 2A) and against M. abscessus grown as biofilm (Fig. 2B).

### Resistance against P4Cs is caused by missense mutations in M. abscessus DNA gyrase.

**FIG 3:**
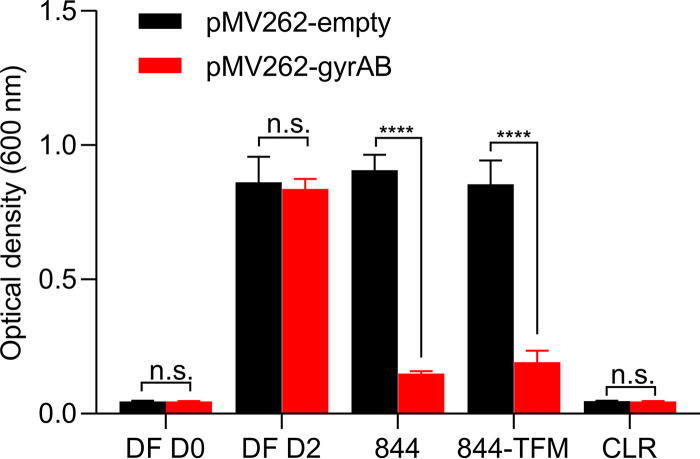
Complementation of P4C-resistant M. abscessus with wild-type *gyrAB*. P4C-resistant M. abscessus subsp. *abscessus* ATCC 19977 884-TFM^r^-1 harboring a D91N mutation in GyrA (Table 2) was transformed with plasmid pMV262 not carrying an insert (pMV262-empty; control) or with pMV262 carrying wild-type *gyrAB* constitutively expressed from the *hsp60* promoter. Cultures were either grown without drug (DF, drug free) or treated with MIC of 844 (8 μM) or 844-TFM (1.5 μM) for 2 days (D2), and growth was measured by OD_600_ determination. Clarithromycin (CLR) treatment at MIC (2 μM) was used as control. The experiments were carried out three times independently, and the results are represented as mean values with the standard deviations displayed as error bars. A two-way ANOVA with Sidak’s multiple comparison test was performed to compare the two groups using GraphPad Prism 8 software.

Based on *in silico* analyses, 844 was proposed to act as an inhibitor of mycobacterial ABC transporters (23). To determine the mechanism of action of 844 experimentally, we isolated spontaneous resistant mutants in M. abscessus subsp. *abscessus* Bamboo on 844-containing agar. Resistant colonies emerged at a frequency of 10^−8^/CFU. Three randomly selected resistant strains were further characterized, showing 2- to more than 8-fold increases in 844 MIC. Whole-genome sequencing, confirmed by targeted sequencing, revealed missense mutations in *gyrA* and *gyrB*, the genes encoding the subunits of DNA gyrase (Table 2). We repeated the mutant selection experiment for 844-TFM using M. abscessus subsp. *abscessus* ATCC 19977, again yielding resistant colonies at a frequency of 10^−8^/CFU. Characterization of five randomly selected resistant colonies revealed 3- to more than 66-fold increased 844-TFM MICs and, again, missense mutations in *gyrA* and *gyrB* (Table 2). MIC determinations of the 844-resistant strains for 844-TFM and of the 844-TFM-resistant strains for 844 revealed cross-resistance of all strains to both compounds (Table 2). To confirm that the observed polymorphisms are indeed causing resistance, one representative resistant strain (M. abscessus subsp. *abscessus* 19977 844-TFM^r^-1), harboring a D91N missense mutation in *gyrA* associated with high-level P4C resistance (Table 2), was complemented with a copy of M. abscessus subsp. *abscessus* ATCC 19977 wild-type (wt) *gyrAB*, which partially restored sensitivity to both 844 and 844-TFM (Fig. 3). These results suggest that missense mutations in M. abscessus DNA gyrase genes cause resistance to P4Cs and that the compounds target this enzyme.

**TABLE 2:** Characterization of 844- and 844-TFM-resistant M. abscessus strains

Strain	MIC (μM)					Mutation^*f*^			
						*gyrA*		*gyrB*	
	844	844-TFM	MXF^*e*^	SPR719^*e*^	CLR^*e*^	Nuc	AA	Nuc	AA
Mab Bamboo WT^*a*^	12	1.5	4	0.8	0.4				
844^r^-1	25	12	2	0.2	0.3	G217C	A73P	A1322C	K441T
844^r^-2	>100	>100	30	0.1	0.3	G271A	D91N		
844^r^-3	90	5	2	0.05	0.4			C1418G	S473W
Mab ATCC 19977 WT^*b*^	8	1.5	3	0.6	2				
844-TFM^r^-1^*c*^	>100	>100	20	0.06	2	G271A	D91N		
844-TFM^r^-2	>100	>100	6	0.04	1	A272G	D91G		
844-TFM^r^-3	40	20	1.5	0.05	2	G136T	V46F		
844-TMF^r^-4	40	6	2	0.3	2			G1422A	M474I
844-TFM^r^-5	25	5	2	0.02	2			G1486T	V496L
MXF^r^-1^*d*^	40	6	100	0.8	2	G286T	D96Y		
MXF^r^-2	15	2	80	0.2	1	G286A	D96N		
MXF^r^-3	15	1	80	0.3	1	A287G	D96G		

a M. abscessus subsp. *abscessus* Bamboo wild type. Used as parental strain for the isolation of spontaneous 844-resistant mutants 844^r^-1 to -3.

b M. abscessus subsp. *abscessus* ATCC 19977 wild type. Used as parental strain for the isolation of spontaneous 844-TFM-resistant mutants 844-TFM^r^-1 to -5.

c Strain used for complementation (Fig. 3), generation of GyrA D91N mutant recombinant DNA gyrase (Fig. 4), and for *recA* promoter DNA damage reporter analysis in P4C-resistant background (Fig. 5).

d M. abscessus subsp. *abscessus* ATCC 19977 was also used to isolate spontaneous moxifloxacin-resistant mutants MXF^r^-1 to -3. High-level MXF resistance causing GyrA D96 mutations (30) caused low-level or no cross-resistance to P4Cs.

e Moxifloxacin (MXF) and SPR719 MICs are included for determination of cross-resistance to other DNA gyrase inhibitors and CLR as negative control.

f Nuc, nucleotide substitution; AA, amino acid substitution.

### P4Cs inhibit activity of recombinant M. abscessus wild-type DNA gyrase but not mutant enzyme harboring a P4C resistance mutation.

To provide direct evidence that the P4Cs indeed target DNA gyrase, we tested whether the molecules inhibit the supercoiling activity of recombinant M. abscessus DNA gyrase. The two DNA gyrase inhibitors, moxifloxacin and SPR719, inhibited supercoiling activity as expected, whereas the negative control, the ribosome inhibitor clarithromycin, did not affect the activity of the enzyme (Fig. 4). Both P4Cs inhibited activity of DNA gyrase (Fig. 4). Consistent with its improved whole-cell activity, the 50% inhibitory concentration (IC_50_) of 844-TFM (1.9 μM) was 2.4-fold lower than the IC_50_ of the parental 844 (4.6 μM) (Fig. 4). To confirm the mechanism of resistance, we tested activity of the P4Cs against recombinant M. abscessus DNA gyrase harboring the resistance mutation D91N in *gyrA* (M. abscessus subsp. *abscessus* ATCC 19977 844-TFM^r^-1) (Table 2; Fig. 3). The mutant version of DNA gyrase was not inhibited by P4Cs (Fig. 4).

**FIG 4:**
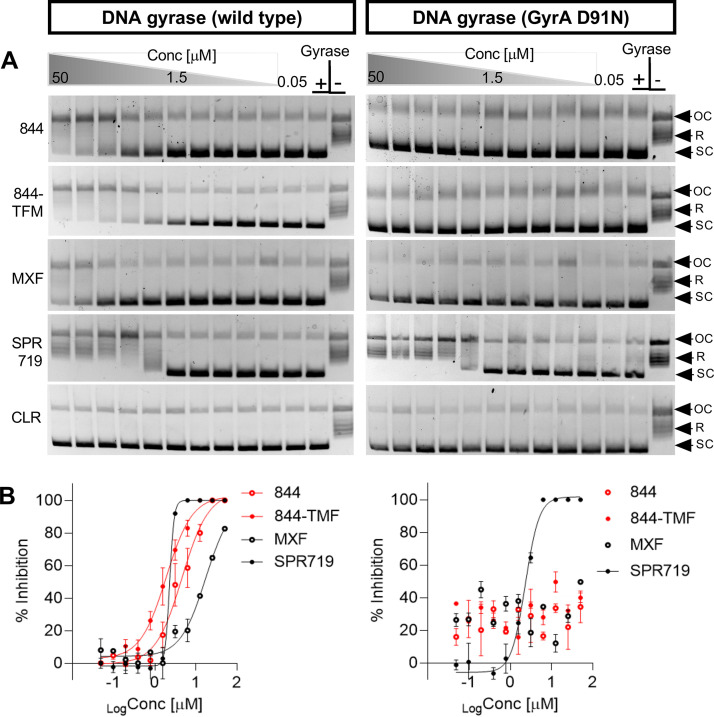
Effect of 844 and 844-TFM on supercoiling activity of M. abscessus wild type and GyrA D91N mutant recombinant DNA gyrase. Relaxed pBR322 plasmid was used as substrate to measure the effect of the P4Cs on the supercoiling activity of wild type or GyrA D91N mutant recombinant DNA gyrase derived from M. abscessus subsp. *abscessus* ATCC 19977 and M. abscessus subsp. *abscessus* ATCC 19977 844-TFM^r^-1, respectively (Table 2). (A) The conversion of relaxed (R) into supercoiled (SC) plasmid was visualized by agarose gel electrophoresis. OC, open circular plasmid; Gyrase −, reaction without added enzyme showing unaltered substrate; Gyrase +, drug-free reaction with added enzyme showing conversion of relaxed plasmid into its supercoiled form. Triangles indicate drug containing reactions with drug concentrations ranging from 50, 25, 12.5, 6.2, 3.1, 1.5, 0.7, 0.39, 0.19, 0.09, to 0.05 μM. Moxifloxacin (MXF) and SPR719 were used as positive and clarithromycin (CLR) as negative controls. The bands presenting supercoiled plasmid were quantified by Invitrogen iBright FL1000 imaging system to determine IC_50_ for the inhibitors. (B) Percent inhibition was calculated in reference to drug-free reactions. IC_50_ values were determined using nonregression model fit of GraphPad Prism 8.0.1 software. 884 and 844-TFM inhibited wild-type DNA gyrase with IC_50_s of 4.6 μM and 1.9 μM. No inhibition of activity was observed when GyrA D91N mutant gyrase was used. MXF and SPR719 inhibited wild-type gyrase with IC_50_s of 17 μM and 2.3 μM, respectively. The D91N mutant version of DNA gyrase was inhibited by SPR719 with the same IC_50_ as the wild-type enzyme. The IC_50_ of MXF was higher than 50 μM for the mutant enzyme. The experiments were repeated three times, independently yielding the same results and a representative example is shown in panel A. Means and standard deviations are shown in panel B.

### P4C resistance causing DNA gyrase mutations show limited cross-resistance to moxifloxacin and no cross-resistance to SPR719.

Taken together, the genetic and biochemical analyses indicate that P4Cs target M. abscessus DNA gyrase, an essential and clinically validated target in mycobacteria (24). The type IIA DNA topoisomerase is a GyrA_2_GyrB_2_ heterotetrameric protein that regulates DNA topology (25). The unwinding of DNA during replication and transcription introduces positive supercoils that, left unaddressed, would affect DNA function. This problem is resolved by DNA gyrase, which introduces negative supercoils into DNA in an ATP-dependent fashion. To do this, the enzyme generates a DNA double-stranded break, passes a segment of double-stranded DNA through the break, and subsequently reseals the DNA molecule (25).

The DNA gyrase inhibitor moxifloxacin (Fig. 1) is a pillar of the treatment of multidrug-resistant tuberculosis (26) and is used less widely for the treatment of macrolide-resistant M. abscessus disease (27, 28). The fluoroquinolone targets the catalytic core of the enzyme comprised of the C-terminal TOPRIM domains of two GyrB subunits and the N-terminal breakage-and-reunion domains of two GyrA subunits (29). Consequently, acquired fluoroquinolone resistance involves missense mutations within these domains (30). P4C resistance mutations (Table 2) are also located in the TOPRIM and breakage-and-reunion domains. Interestingly, D91 missense mutations causing high-level P4C resistance have been reported to also confer resistance to moxifloxacin in M. tuberculosis (31). To determine whether the P4C resistance mutations in M. abscessus confer cross-resistance to moxifloxacin, we measured the MICs of moxifloxacin for the P4C-resistant strains. The strains harboring D91 missense mutations in *gyrA* showed low level cross-resistance with a 2- to 8-fold increase in MIC (Table 2). None of the other P4C resistance mutations altered the fluoroquinolone MIC (Table 2).

A novel DNA gyrase inhibitor, SPR719 (Fig. 1), is in clinical development for the treatment of mycobacterial lung diseases. This benzimidazole acts as an inhibitor of the ATPase activity of DNA gyrase located in the N-terminal domain of GyrB (32). SPR719-resistant mutants in M. tuberculosis have been mapped to the ATPase domain (32). As expected, P4C resistance mutations did not cause cross-resistance to SPR719 (Table 2). Interestingly, several P4C-resistant mutations conferred hypersusceptibility to SPR719. The mechanistic basis for this phenotype remains to be determined.

Thus, P4C-resistant mutations caused limited or no cross-resistance to moxifloxacin or SPR719, suggesting a novel on-target mechanism of this new DNA gyrase inhibitor.

### P4Cs trigger induction of *recA* DNA damage reporter in wild-type but not in P4C-resistant M. abscessus.

**FIG 5:**
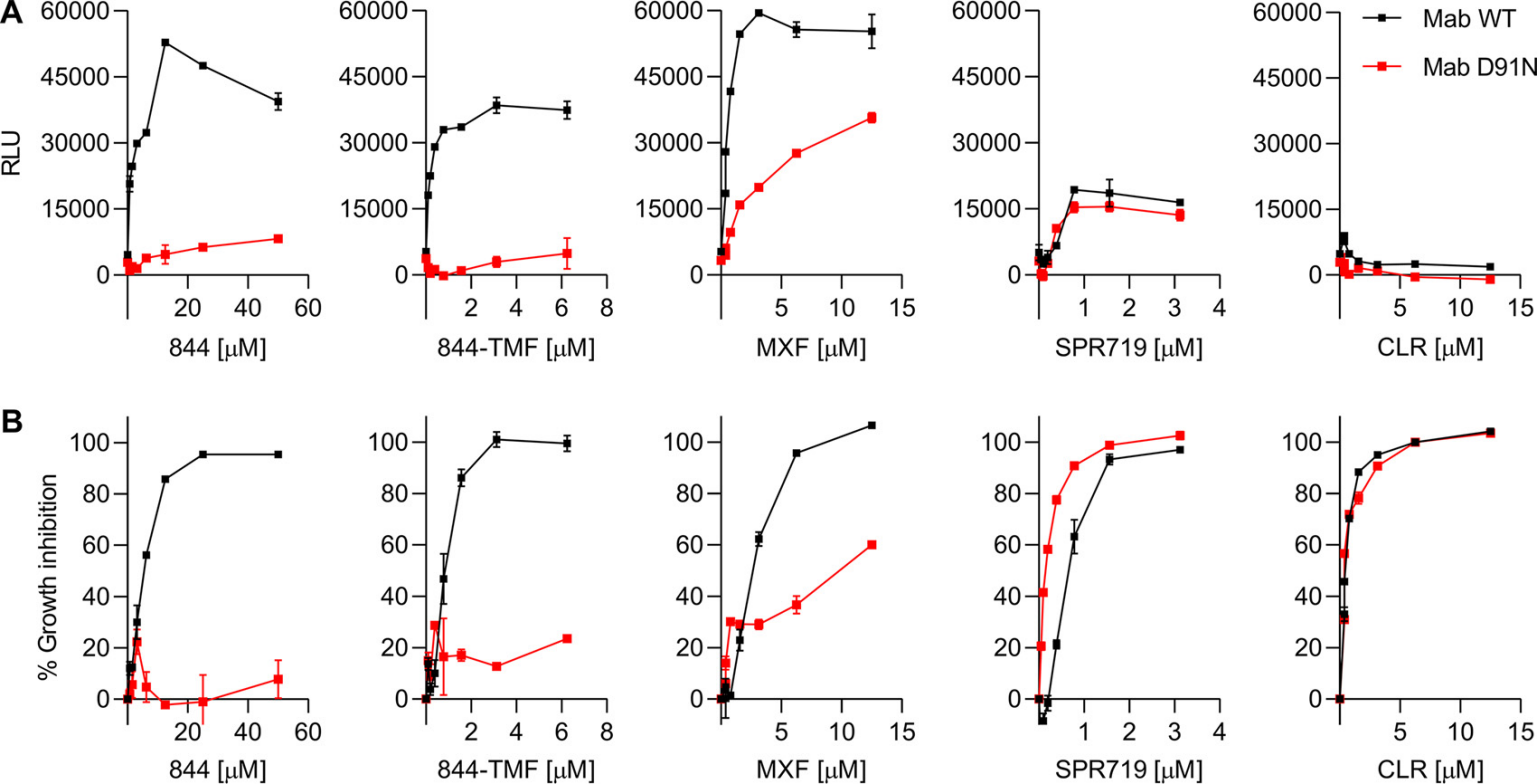
Effect of 844 and 844-TFM on *recA* promoter-driven LuxCDABE operon expression in wild-type and P4C-resistant *gyrA* D91N mutant M. abscessus. (A) Exponentially growing cultures of wild-type M. abscessus subsp. *abscessus* ATCC 19977 (black, Mab WT) or M. abscessus subsp. *abscessus* ATCC 19977 884-TFM^r^-1 (Table 2), carrying the D91N allele of *gyrA* (red, Mab D91N), both harboring a LuxCDABE expressed under the control of the DNA damage inducible *recA* promoter, were treated with increasing concentration of 844 or 844-TFM for 4 h, and relative luminescence (RLU) was measured as a readout for *recA* promoter activity. The experiments were also carried out for moxifloxacin (MXF) and SPR719 for comparison and for clarithromycin (CLR) as negative control. (B) Growth inhibitory effects of the drugs used in panel A determined via OD_600_ measurement after treatment of cultures for 3 days. (A, B) experiments were carried out three times independently, and the results are represented as mean values with the standard deviations displayed as error bars.

Fluoroquinolones arrest DNA gyrase—DNA complexes in their double strand broken state. This mechanism of action results in DNA damage, which contributes to the bactericidal activity of the class (33). Similar to moxifloxacin, P4Cs are bactericidal and resistance mutations map to the catalytic core of DNA gyrase. To determine whether P4Cs also cause DNA damage, we constructed a DNA damage reporter strain by introducing the DNA damage inducible *recA* promoter controlling expression of the bioluminescence LuxCDABE operon into M. abscessus subsp. *abscessus* ATCC 19977. The positive control moxifloxacin strongly induced *recA* promoter-dependent reporter expression (Fig. 5), similar to what has been described for M. tuberculosis (34, 35). SPR719, inhibiting gyrase’s ATPase activity, caused only a weak increase, and treatment of M. abscessus with the protein synthesis inhibitor clarithromycin as negative control did not cause any increase in reporter expression (Fig. 5). Similar to moxifloxacin, treatment of reporter cultures with P4Cs strongly induced expression of the reporter gene (Fig. 5), suggesting that the compounds damage bacterial DNA. If P4C-mediated *recA* promoter induction is due to interaction of the compounds with DNA gyrase, induction should not occur in P4C-resistant *gyrAB* mutant background. To test this prediction, we introduced the *recA* reporter operon into the P4C-resistant M. abscessus subsp. *abscessus* ATCC 19977 844-TFM^r^-1 strain harboring the D91N allele of *gyrA* (Table 2; Fig. 3 and 4). In this background, induction of *recA* promoter activity was strongly reduced, suggesting that the increase of *recA* promoter activity in the wild-type background is DNA gyrase dependent (Fig. 5). Together, these results suggest that inhibition of DNA gyrase by P4Cs results in DNA damage.

### *In vivo* and *in vitro* pharmacokinetic properties of 844-TFM.

**FIG 6:**
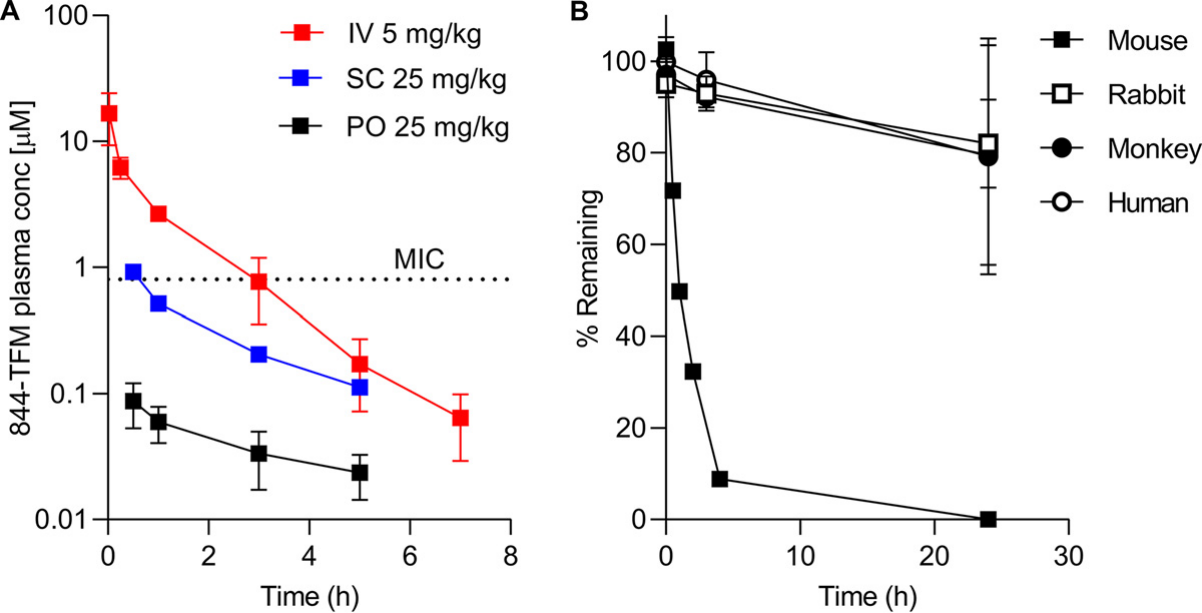
Concentration-time profile and plasma stability of 884-TFM. (A) Concentration-time profile of 884-TFM following a single intravenous (i.v.), subcutaneous (s.c.), or oral (p.o.) dose of 5, 25, and 25 mg/kg, respectively, to CD-1 mice. MIC indicates 884-TFM MIC (=3.5 μM) (Table 1) for M. abscessus subsp. *abscessus* K21, the strain we employ in our murine infection model to determine efficacy of drugs (43). (B) Compound stability in mouse, rabbit, monkey, and human plasma over 24 h. For panels A and B, mean and standard deviations (*n* = 3) are shown.

To identify the pharmacokinetic (PK) liabilities of 884-TMF, we determined its concentration-time profile in mice and measured basic PK properties in established *in vitro* assays. 884-TFM was not orally bioavailable in mice with a calculated bioavailability of 0.05 to 0.2% (Fig. 6A and Table 3). To determine whether this was due to poor solubility, low permeability, or rapid metabolism, these properties were evaluated in standard *in vitro* PK assays (Table 3). 844-TFM exhibited adequate solubility at both pH 2.0 (simulated gastric fluid) and pH 7.4 (standard physiological conditions). In the parallel artificial membrane permeability assay (PAMPA), 844-TFM showed modest permeability with a logPe of −5.7 cm/s (logPe of −5.0 cm/s is considered the border between high and low permeability). In mouse liver microsomes, 844-TFM was highly unstable with a half-life of 5 min and a high rate of clearance (Table 3), nearly as high as the mouse hepatic blood flow of 90 ml/min/kg (36), predicting an extraction ratio of ∼1, consistent with rapid first-pass liver metabolism and in line with poor oral bioavailability. To circumvent first-pass metabolism, we administered 844-TFM via the subcutaneous route to CD-1 mice, leading to improved exposure compared to that of the oral route (Fig. 6A). However, the compound was still rapidly eliminated, and bioavailability remained modest at 3%. Indeed, 844-TFM was rapidly degraded in mouse plasma (Fig. 6B). Identification of the two major breakdown products in mouse plasma revealed cleavage of the central amide bond of 844-TFM (see Fig. S1 and S2 in the supplemental material). Interestingly, the compound was markedly more stable in rabbit, monkey, and human plasma, with approximately 80% remaining after 24 h at 37°C (Fig. 6B). Taken together, characterization of the PK properties of 844-TFM revealed limited permeability and metabolic instability as the major liabilities of the lead compound in the mouse model.

**TABLE 3:** *In vitro* and *in vivo* pharmacokinetic properties of 844-TFM

Pharmacokinetic property^*a*^	Value
Solubility (μM)	
pH 2	446.5
pH 7.4	390.0
PAMPA, pH 7.4	
Mean Pe (10^−6^cm/s)	2.0
LogPe	−5.7
Mouse Hepatic Microsomes Stability (*in vitro*)	
k*_el_* (min^−1^)	0.1
*t*_1/2_ (min)	5.2
*In vitro* CL_int_ (μl/min/mg protein)	267.3
CL_int_ (ml/min/kg)	1,052.4
CL_hepatic_ (ml/min/kg)	82.9
Mouse pharmacokinetic parameters (*in vivo*)^*b*^	
Intravenous administration	
AUC i.v. 5 mg/kg (h · ng/ml)	5,627 (2,181)
elimination half-life (h)	1.03 (0.17)
*k*_el_ (h^−1^)	0.69 (0.12)
*V* (liter/kg)	0.811 (0.221)
Clearance (ml/kg · h)	638 (231)
Other routes of administration	
AUC p.o. 25 mg/kg (ng · h/ml)	58 (54)
AUC s.c. 25 mg/kg (ng · h/ml)	1,168 (593)
p.o. bioavailability (%)	0.15 (0.14)
s.c. bioavailability (%)	3.0 (1.5)

a PAMPA, parallel artificial membrane permeability assay; Pe, effective permeability coefficient; *k*_el_, elimination rate constant; *t*_1/2_, half-life; CL_int_, intrinsic clearance; CL_hepatic_, hepatic clearance; AUC, area under the concentration-time curve; *V*, volume of distribution; i.v., intravenous; p.o., oral; s.c., subcutaneous.

b Average (SD).

### Conclusion.

Fluoroquinolones are used successfully as second-line agents for the treatment of multidrug-resistant tuberculosis (37). Variable *in vitro* susceptibilities due to (unknown) intrinsic resistance mechanisms limit the therapeutic utility of this drug class against M. abscessus (5). Thus, moxifloxacin is used only rarely as a second-line drug for the treatment of macrolide-resistant infections, and there is no effective DNA gyrase inhibitor available for the treatment of M. abscessus disease. Recently, the benzimidazole SPR719, an inhibitor of ATPase activity of the mycobacterial DNA gyrase, entered early clinical development for tuberculosis and NTM lung diseases, bringing new hope for patients suffering from mycobacterial infections (38). Here, we have identified a novel class of mycobacterial DNA gyrase inhibitors with attractive bactericidal and antibiofilm activity against M. abscessus complex. We characterized the PK properties of the lead compound to enable medicinal chemistry programs. A novel DNA gyrase inhibitor would be a welcomed addition to the anti-M. abscessus drug pipeline.

## MATERIALS AND METHODS

### Bacterial strains, culture media, and drugs.

M. abscessus subsp. *abscessus* ATCC 19977, harboring the inducible clarithromycin resistance-conferring *erm*(41) T28 sequevar (39), was purchased from the American Type Culture Collection. M. abscessus subsp. *bolletii* CCUG 50184T, harboring the inducible clarithromycin resistance-conferring *erm*(41) T28 sequevar (40), and M. abscessus subsp. *massiliense* CCUG 48898T, harboring the nonfunctional *erm*(41) deletion sequevar (41), were purchased from the Culture Collection University of Goteborg. M. abscessus subsp. *abscessus* Bamboo (42) was provided by Wei Chang Huang (Taichung Veterans General Hospital, Taichung, Taiwan). M. abscessus subsp. *abscessus* K21 (43) was provided by Sung Jae Shin (Department of Microbiology, Yonsei University College of Medicine, Seoul, South Korea) and Won-Jung Koh (Division of Pulmonary and Critical Care Medicine, Samsung Medical Center, Seoul, South Korea). The clinical M isolates of M. abscessus (44) were provided by Jeanette W. P. Teo (Department of Laboratory Medicine, National University Hospital of Singapore).

Mycobacterium abscessus was grown in complete Middlebrook 7H9 broth (271310; BD Difco, Spark, MD, USA) supplemented with 0.05% Tween 80, 0.2% glycerol, and 10% albumin-dextrose-catalase. 7H10 agar (262710; BD Difco, Sparks, MD, USA) was used as solid medium.

844 was obtained from the Medicines for Malaria Venture’s (Geneva) compound archive. 844-TFM was synthesized as described below. Moxifloxacin (MXF, SML1581) and clarithromycin (CLR, C9742) were purchased from Sigma-Aldrich, USA. SPR719 (HY-12930) was purchased from MedChemExpress LLC, USA. MXF, SPR719, and CLR were dissolved in 100% dimethyl sulfoxide (DMSO) (MP Biomedicals, USA) at 10 mM. 844 and 844-TFM were dissolved in ethanol (BP2818100; Fisher Scientific, USA) at 5 mM and 10 mM, respectively. All compounds were stored in aliquots at −20°C until use.

### Chemicals and physical methods.

Starting materials were purchased and used as received. Solvents were of reagent grade and distilled before use. *N*-(6-methoxy-1,5-naphthyridin-4-yl)-4-piperidinecarboxamide was prepared according to the literature (45, 46). The melting point (uncorrected) was determined on a Boetius melting-point apparatus (VEB Kombinat NAGEMA, Dresden, GDR). ^1^H and ^13^C nuclear magnetic resonance (NMR) spectra were recorded at room temperature on an Agilent Technologies VNMRS 500 NMR spectrometer. The residual solvent signals of methanol-*d*_4_ (δ_1H_ = 3.31 ppm; δ_13C_ = 49.00 ppm) were used to reference the spectra (abbreviations, s = singlet, d = doublet, t = triplet, q = quartet, td = triplet of doublets, m = multiplet). The mass spectrum was recorded on a Q Exactive Plus Orbitrap mass spectrometer (Thermo Scientific, Bremen, Germany) using methanol as solvent.

### Synthesis of 844-TFM.

A mixture of *N*-(6-methoxy-1,5-naphthyridin-4-yl)-4-piperidinecarboxamide (50 mg, 0.17 mmol), triethylamine (35 μl, 0.26 mmol, 1.5 eq), and 1-(2-bromoethyl)-4-(trifluoromethyl)benzene (58 μl, 0.34 mmol, 2 eq) in dimethylformamide (DMF) (5 ml) was stirred at 50°C for 16 h. The reaction was quenched by the addition of water (20 ml). The mixture was extracted with ethyl acetate (3 × 10 ml), and the combined organic phases were washed with water (10 ml) and brine (10 ml). The solution was dried over Na_2_SO_4_, filtered, and evaporated under reduced pressure. The residue was chromatographed on silica gel with methanol/ethyl acetate (0 to 5%) to afford the product as a white solid (40 mg, 0.09 mmol, 51%); M.p. 433-435 K; ^1H^ NMR (500 MHz, methanol-*d*_4_) δ 8.61 (d, *J* = 5.2 Hz, 1H), 8.50 (d, *J *= 5.2 Hz, 1H), 8.19 (d, *J* = 9.1 Hz, 1H), 7.58 (d, *J* = 8.0 Hz, 2H), 7.43 (d, *J* = 8.0 Hz, 2H), 7.26 (d, *J* = 9.1 Hz, 1H), 4.16 (s, 3H), 3.14 (dt, *J* = 12.1, 3.5 Hz, 2H), 2.96 to 2.91 (m, 2H), 2.74 to 2.66 (m, 3H), 2.27 (td, *J *= 11.8, 2.5 Hz, 2H), 2.09 (d, *J* = 12.6 Hz, 2H), 1.93 (qd, *J* = 11.7, 4.3 Hz, 2H) (see Fig. S3 in the supplemental material); ^13^C NMR (126 MHz, methanol-*d*_4_) δ 174.7, 161.8, 148.3, 144.7, 144.7, 140.4, 140.3, 139.2, 132.0, 129.0, 128.2, 128.0, 125.5, 124.9 (q, *J* = 3.9 Hz), 124.9, 124.9, 124.8, 117.1, 110.8, 59.6, 53.3, 52.4, 43.5, 32.4, 28.1, 19.3 (see Fig. S3); electrospray ionization-high-resolution mass spectrometry (ESI-HRMS): *m/z* [M+H]^+^ calculated for C_24_H_26_F_3_N_4_O 459.2007, found 459.1995.

### Determination of MICs.

MIC was determined using the broth microdilution method in 96-well plates as described previously (47). Briefly, a 10-point 2-fold serial dilution of compounds was performed in 96-well plates (Costar 3370; Corning, USA) starting at twice the desired highest concentration. Exponentially growing M. abscessus cultures (optical density at 600 nm [OD_600_] = 0.4 to 0.8) were adjusted to a density of OD_600_ = 0.01 in Middlebrook 7H9 broth (Becton, Dickinson). One hundred microliters of the bacterial suspension was seeded onto the 96-well plates containing 100 μl of the serially diluted compounds to give a final volume of 200 μl in each well with the final OD_600_ of 0.005. The plates were sealed with parafilm, placed onto wet paper towels in a lock-lock box and incubated for 3 days at 37°C with orbital shaking at 90 rpm. Absorbance at 600 nm was measured using a TECAN Infinite Pro 200 plate reader after resuspension. Absorbance values at day 3 were subtracted from the day 0 readout. Percent growth inhibition was calculated by dividing the absolute absorbance value of treated cells with untreated control and multiplying by 100. CLR was included in all MIC experiments as a positive control to monitor assay reproducibility. MIC was defined as 90% of growth inhibition relative to untreated controls.

### Time-concentration kill assay.

To determine the bactericidal activity of 844 and 844-TFM, exponentially growing M. abscessus subsp. *abscessus* ATCC 19977 cultures (OD_600_ = 0.4 to 0.8) were diluted to a final OD_600_ of 0.005, and 1 ml of the culture suspension was exposed to 1×, 4×, and 8× MIC of 844 (MIC = 8 μM), 844-TFM (MIC = 1.5 μM), MXF (MIC = 3 μM), SPR719 (MIC = 0.6 μM), and CLR (MIC = 2 μM) in 14-ml vented, round-bottom tubes (150268; Fisher Scientific, USA) and incubated at 37°C on an orbital shaker at 200 rpm. At 0, 2, and 3 days, tubes were vortexed, and 20 μl of the cultures was transferred to round-bottom 96-well plates containing 180 μl of phosphate-buffered saline (PBS) (10010023; Thermo Fisher, USA) with 0.025% Tween 80 (PBS/Tween 80) for 10-fold serial dilution. To prevent compound carryover effects, we plated out serially diluted samples onto 7H10 agar supplemented with 0.4% activated charcoal (C9154; Sigma-Aldrich, USA) for all time points as described (48). CFU were enumerated after 4 days of incubation at 37°C. A two-way analysis of variance (ANOVA) multiple comparison test was performed using GraphPad Prism 8 software to compare treated groups with untreated day 0 CFU.

### Determination of antibiofilm activity.

The antibiofilm activity of 844 and 844-TMF was determined employing the MBEC 96-well Biofilm assay kit (19111; Innovotech, Edmonton, AB, Canada) as described previously (15) with minor modifications. Briefly, exponentially growing M. abscessus subsp. *abscessus* ATCC 19977 cultures were spun down at 3,200 × *g* for 10 min and washed with 7H9 broth without Tween 80 (‘7H9’). Bacteria were resuspended in ‘7H9’ to an OD_600_ of 0.0125, and 150 μl was dispensed into the wells of Innovotech 96-well plates. Pegs (protruding from the specialized lids of the Innovotech multiwell plates) were inserted into the bacterial suspensions in the wells, and the cultures were grown 24 h with shaking at 110 rpm at 37°C to allow attachment and growth of the bacteria on the surface of the pegs. Then, the lids with the pegs were transferred to a new multititer plate containing 150 μl fresh ‘7H9’ broth (without bacteria; time zero) (Fig. 2B) with no drug or with 1×, 4×, or 8× MIC of 844, 844-TFM, MXF, SPR719, or CLR and incubated for 0, 2, and 3 days. Biofilm growth on the pegs was measured by CFU determination. The pegs were washed in 200 μl ‘7H9’ medium, removed, and placed in 1.7-ml microcentrifuge tubes (87003-294; VWR, Radnor, PA, USA) containing 500 μl PBS/Tween 80 (0.025%). The tubes were vortexed at 2,000 rpm for 90 s to detach and suspend the bacteria from the pegs before samples were serially diluted and plated onto 7H10 agar supplemented with 0.4% activated charcoal to determine CFU/peg. A two-way ANOVA multiple comparison test was performed using GraphPad Prism 8 software to compare treated groups with untreated day 0 CFU.

### Selection of spontaneous resistant mutants, whole-genome, and targeted sequencing.

Spontaneous resistant mutants against 844 and 844-TFM were selected using M. abscessus subsp. *abscessus* Bamboo and M. abscessus subsp. *abscessus* ATCC 19977 as described previously (49). Briefly, 10^7^ to 10^9^ CFU of exponentially growing cultures were plated on 7H10 agar containing 8× MIC of 844 or 844-TFM (or 32× MIC of MXF for the selection of MXF-resistant strain). The plates were sealed with parafilm and incubated for 7 days at 37°C. Apparent resistant colonies were purified and confirmed by restreaking on 7H10 agar containing the same concentration used for selection of resistant colonies. Genomic DNA was extracted as described previously using the phenol-chloroform method (50). Whole-genome sequencing, including library construction and bioinformatics analyses, was performed by Genewiz (Genewiz, Inc., South Plainfield, NJ, USA). Polymorphisms detected in *gyrA* (MAB_0019) and *gyrB* (MAB_0006) by whole-genome sequencing were confirmed by Sanger sequencing (Genewiz, Inc., South Plainfield, NJ, USA) using primers for *gyrA* (Fw-*gyrA* 5′-GCATCTAAAGCCGCTGAGAACG-3′; Rv-*gyrA* 5′-GGTCCACGGGGCGTTCGTTTGC-3′) and *gyrB* (Fw-*gyrB* 5′-CTGAAACTAGGTGCCGTGGGTGC-3′; Rv-*gyrB* 5′-CTTGAACTACCTGGGCGGGTTACC-3′).

### Complementation of P4C-resistant M. abscessus.

To complement the P4C-resistant M. abscessus subsp. *abscessus* ATCC 19977 844-TFM^r^-1 strain harboring a D91N allele of *gyrA* (Table 2), wild-type *gyrA* and *gyrB* were cloned into pMV262 (51) and expressed from the plasmid’s constitutive *hsp60* promoter as described previously (50). The coding sequences of *gyrB* and *gyrA* were amplified from plasmid pET28a+gyrBA wt, which had been constructed to express recombinant wild-type DNA gyrase (see below) using primers Fw-*gyrBA* (HindIII, underlined) 5′-gcgaagcttCATCATCATCATCATCACGCTGCCCAGAAGAAGAGTG-3′ and Rv-*gyrBA* (HindIII, underlined) 5′-gcgaagcttTTACTCGCCTGCGGTCGTG-3′ (lowercase sequences indicate added sequences with restriction enzyme recognition sites underlined) and Phusion high-fidelity DNA polymerase (F530S; Fisher Scientific, USA). To allow in-frame cloning of the *gyrBA* coding sequences to the N-terminal *hsp60* coding sequence via pMV262’s HindIII site, pMV262’s DraI-HindIII segment (carrying the *hsp60* promoter and the *hsp60* N-terminal coding sequence) was replaced by a shortened DraI-HindIII fragment amplified using the primers Fw-*hsp60* (DraI, underlined) 5′-cgctttaaaTCTAGAGGTGACCACAACG-3′ and Rv-*hsp60* (HindIII, underlined) 5′-gcgaagcttCGCAATTGTCTTGGCCATTGC-3′) and pMV262 as template. The HindIII-digested *gyrBA* insert was ligated into HindIII-digested modified pMV262 plasmid and transformed into Escherichia coli DH5α. pMV262-p*hsp60*-gyrBA harboring transformants was identified by PCR, and the plasmid was electroporated M. abscessus subsp. *abscessus* ATCC 19977 844-TFM^r^-1 as described (52). Transformants were selected on 7H10 agar containing 400 μg/ml kanamycin and verified by PCR to harbor pMV262-p*hsp60*-gyrBA. M. abscessus subsp. *abscessus* ATCC 19977 844-TFM^r^-1 harboring pMV262-p*hsp60*-gyrBA or modified pMV262-p*hsp*60 plasmid without *gyrBA* insert (pMV262-empty) were treated with 844 or 844-TFM or the negative control CLR at 1× MIC in 14-ml vented, round-bottom tubes (150268; Fisher Scientific, USA) and incubated at 37°C on an orbital shaker at 200 rpm. After 2 days of incubation, tubes were vortexed and 200 μl of the samples from each tube transferred into a clear bottom 96-well plate for absorbance measurement at 600 nm using a TECAN Infinite Pro 200 plate. A two-way ANOVA with Sidak's multiple comparison test was performed using GraphPad Prism 8 software to compare the two groups.

### Cloning, expression, and purification of recombinant M. abscessus DNA gyrases.

To clone and express recombinant M. abscessus DNA gyrase *gyrA* and *gyrB* (MAB_0019, MAB_0006), we adapted the strategy previously used for expression of recombinant M. tuberculosis DNA gyrase (53). The coding regions for residues 2 to 675 of GyrB and 2 to 839 of GyrA were amplified from genomic DNA of wild-type M. abscessus subsp. *abscessus* ATCC 19977 using primers Fw-*gyrA* (NheI, underlined) 5′-gcagcagctagcATGACTGACACAACGCTGCCCCCC-3′, Rv-*gyrA* (HindIII, underlined) 5′-tgctgcaagcttTTACTCGCCTGCGGTCGTGCCGGC-3′, Fw-*gyrB* (NdeI, underlined) 5′-gcagcacatatgGCTGCCCAGAAGAAGAGTGCCAAG-3′, and Rv-*gyrB* (NheI, underlined) 5′-tgctgcgctagcTACGTCGAGGAAGCGCACGTCC-3′. The fusion GyrB and GyrA with an alanine-serine linker was cloned into pET-28a(+) containing an N-terminal hexahistidine tag using the restriction enzymes NdeI (R0111S), NheI (R3131S), and HindIII (R3104M) from New England BioLabs, resulting in the plasmid pET28a+gyrBA wt. For expression of recombinant DNA gyrase harboring a D91N substitution in GyrA, the analogous plasmid pET-28a+gyrBA^D91N^ was generated via gene synthesis and cloning using Genewiz’s gene synthesis service.

Wild type and GyrA D91N mutant DNA gyrase were expressed and purified as described (53). Proteins were overexpressed in E. coli BL21(DE3) pLysE by growing the cells at 37°C until early log phase and then treating the cultures with 1 mM isopropyl-β-d-thiogalactopyranoside for 3 h at 30°C (I5502; Sigma-Aldrich, USA). Cells were harvested by centrifugation and resuspended in buffer A500 (20 mM Tris-HCl, pH 7.9, 500 mM NaCl, 5 mM imidazole, pH 8.0) with protease inhibitor cocktail, 4-(2-aminoethyl)benzenesulfonyl fluoride hydrochloride (AEBSF), aprotinin, bestatin, E64, leupeptin, and pepstatin A (Halt protease inhibitor cocktail [100×], 78429; Thermo Scientific, USA). For protein purification, cells were sonicated and centrifuged (8,000 rpm, 4°C), and the clarified lysate was passed over an Ni gravity column (IMAC nickel resin, 1560131; Bio-Rad, USA) preequilibrated with the buffer A500. The column was washed with 10 column volumes of buffer A500. The protein was eluted with buffer B500 (20 mM Tris-HCl, pH 7.9, 500 mM NaCl, 250 mM imidazole, protease inhibitors), and the fractions were pooled and dialyzed overnight at 4°C against buffer A500. For the biochemical assays, the proteins were concentrated using Pierce protein concentrator PES (10K MWCO, 5 to 20 ml; Thermo Scientific, USA), aliquoted, and stored at −20°C.

### *In vitro* DNA gyrase inhibition assay.

The mycobacterial DNA gyrase inhibition assay was performed using the kit from Inspiralis Limited (MTS002; Norwich, UK) as described by the suppliers using the generated recombinant DNA gyrases from M. abscessus instead of the recombinant M. tuberculosis DNA gyrase supplied by the kit. The assay was carried out in a 30-μl final volume, containing 50 mM HEPES KOH (pH 7.9), 6 mM magnesium acetate, 4 mM dithiothreitol (DTT), 1 mM ATP, 100 mM potassium glutamate, 2 mM spermidine, 0.05 mg/ml bovine serum albumin, 40 nM either wild-type or GyrA D91N mutant DNA gyrase, and 0.5 μl (1 μg/μl) of relaxed pBR322 DNA. The reaction mixture was incubated for 30 min at 37°C, and then stopped by adding 30 μl of chloroform/isoamyl alcohol (24:1) followed by 30 μl of STEB (40% sucrose, 100 mM Tris-HCl [pH 8.0], 100 mM EDTA, and 0.5 mg/ml bromophenol blue) solution. The tubes were briefly vortexed and centrifuged for 1 min, and 20 μl of the aqueous (upper blue) phase was loaded onto a 1% (wt/vol) agarose gel. The gel was run for 2 h at 75 V and stained with 1 μg/ml ethidium bromide in water. After destaining with water for 10 min, images were taken and analyzed using Invitrogen iBright FL1000 imaging system. To determine IC_50_ values, the intensity of the bands was measured and compared to the drug-free reaction using iBright analysis software. The IC_50_ values were determined using nonregression model fit of GraphPad Prism 8.0.1 software.

### *recA* DNA damage report assay.

To assess whether compounds cause DNA damage, luminescence of M. abscessus cultures harboring a LuxCDABE reporter expressed under the control of the *recA* promoter was measured. A *recA*-LuxCDABE reporter plasmid was constructed by replacing the NotI-EcoRI *hsp60* promoter fragment of pMV306*hsp60*-LUX (54) (Addgene, 26159) with the M. abscessus subsp. *abscessus* ATCC 19977 *recA* promoter (34). The M. abscessus *recA* promoter was amplified with primers Fw-P*recA* (NotI, underlined) 5′-gcgcggccgcGTTGGGGGAACCGCGTTAC-3′ and Rv-P*recA* (EcoRI, underlined) 5′-ccggaattcGGTGTTCTCCGTTTCGTCG-3′ using Phusion high-fidelity DNA polymerase (F530S; Fisher Scientific, USA). The resulting amplicon was digested with NotI (no. R31189L; New England BioLabs) and EcoRI (no. R3101S; New England BioLabs), gel purified (Qiagen, Hilden, Germany), and ligated into NotI-EcoRI-digested pMV306*hsp*60-LUX, resulting in the reporter plasmid pMV306*recA*-LUX (54). The ligation product was transformed into E. coli DH5α and plated on LB agar medium containing 25 μg/ml kanamycin. The plasmid was verified by PCR and transformed into wild-type M. abscessus subsp. *abscessus* ATCC 19977 and the P4C-resistant M. abscessus subsp. *abscessus* ATCC 19977 844-TFM^r^-1 strain harboring a D91N allele of *gyrA* (Table 2) via electroporation (52). To measure the effect of compounds on *recA* promoter activity, exponentially growing reporter cultures (OD_600_ = 0.4 to 0.8) were adjusted to an OD_600_ of 0.1 and treated with various concentrations of drugs. Luminescence intensity was measured using a TECAN Infinite Pro 200 plate reader at 250 ms integration time with automatic attenuation at time zero and after 4 h at 37°C. Data analysis was performed using GraphPad Prism 8 software after subtracting the luminescence at time zero.

### *In vitro* pharmacokinetic analyses.

Kinetic solubility, parallel artificial membrane permeability assay (PAMPA) permeability, and stability in the mouse liver microsome assay were performed by BioDuro (Shanghai, China) according to standard protocols.

### *In vivo* mouse pharmacokinetics.

All animal experiments were approved by the Center for Discovery and Innovation, Institutional Animal Care and Use Committee, and were conducted in compliance with their guidelines. Female CD-1 mice were weighed and received a single dose of 884-TFM via the intravenous (i.v.) (5 mg/kg of body weight), oral (p.o.) (25 mg/kg), or subcutaneous (s.c.) (25 mg/kg) dosing route. The compound was formulated as a solution in 5% *N*,*N*-dimethylacetamide (DMA)/95% Milli-Q water vehicle. Blood samples were serially collected via the tail snip from each individual mouse at 0.017, 0.25, 1, 3, 5, and 8 h postdose following i.v. dosing and at 0.5, 1, 3, and 5 h postdose following p.o. and s.c. dosing. Blood (50 μl) was collected in capillary Microvette K_2_EDTA tubes (16.444.100; Sarstedt, Inc.) and kept on ice prior to centrifugation at 1,500 × *g* for 5 min. The supernatant (plasma) was transferred into a 96-well plate and stored at −80°C.

### HPLC-MS analysis.

Liquid chromatography-tandem mass spectrometry (LC-MS/MS) analysis was performed on a Sciex Applied Biosystems Qtrap 6500+ triple-quadrupole mass spectrometer coupled to a Shimadzu Nexera 2 high-pressure liquid chromatography (HPLC) system to quantify each drug in plasma. Neat 1 mg/ml DMSO stocks for 844-TFM were serial diluted in 50:50 acetonitrile (ACN)/water to create standard curves and quality control (QC) spiking solutions. Standards and QCs were created by adding 10 μl of spiking solutions to 90 μl of drug-free plasma (CD-1 K_2_EDTA Mouse; Bioreclamation IVT). Twenty microliters of control, standard, QC, or study sample were added to 200 μl of ACN/methanol (MeOH) 50:50 protein precipitation solvent containing internal standard (10 ng/ml verapamil). Extracts were vortexed for 5 min and centrifuged at 4,000 rpm for 5 min. One hundred microliters of supernatant was transferred for HPLC-MS/MS analysis and diluted with 100 μl of Milli-Q deionized water.

Chromatography was performed on an Agilent Zorbax SB-C_8_ column (2.1 × 30 mm; particle size, 3.5 μm) using a reverse-phase gradient. Milli-Q deionized water with 0.1% formic acid was used for the aqueous mobile phase and 0.1% formic acid in ACN for the organic mobile phase. Multiple-reaction monitoring of precursor/product transitions in electrospray positive-ionization mode was used to quantify the analytes. The following multiple reaction monitoring (MRM) transitions were used for 844-TFM (459.10/133.00) and verapamil (455.4/165.2). Sample analysis was accepted if the concentrations of the quality control samples were within 20% of the nominal concentration. Data processing was performed using Analyst software (version 1.6.2; Applied Biosystems Sciex).

### Plasma stability analysis and metabolite identification.

The plasma stability assays were carried out using plasma from female CD-1 mice, New Zealand white rabbits, rhesus monkeys, and humans containing K_2_EDTA anticoagulant (Bioreclamation). Stability samples consisted of 5 μl of stock compound solution in 50:50 ACN/water and 95 μl of plasma to a final concentration of 1 μg/ml. The samples were incubated at 37°C with shaking; 10 μl of plasma was removed at each time point and combined with 100 μl of ACN/methanol 50:50 protein precipitation solvent containing internal standard (10 ng/ml verapamil). Chromatography for metabolite identification was performed the same as specified for pharmacokinetic sample analysis. Full scan total ion chromatograms (TIC) of plasma extracts were acquired using a Q Exactive high-resolution mass spectrometer (QE-HRMS) at 70,000 mass resolution. Thermo Fisher Compound Discoverer software was used to assist in identifying the 844-TFM metabolites from the QE-HRMS TIC mass spectrum. Figure S2 in the supplemental material illustrates the extracted ion chromatograms (XIC) of 844-TFM and the metabolites using 5 ppm mass accuracy before and after 12 h of incubation in mouse plasma.

## References

[1] Tortoli E. 2019. The taxonomy of the genus mycobacterium, p 1–10. *In* Velayati AA, Farnia P (ed), Nontuberculous mycobacteria (NTM). Academic Press, New York, NY.

[2] Vinnard C, Longworth S, Mezochow A, Patrawalla A, Kreiswirth BN, Hamilton K. 2016. Deaths related to nontuberculous mycobacterial infections in the United States, 1999–2014. Ann Am Thorac Soc 13:1951–1955. 10.1513/AnnalsATS.201606-474BC. 27607541 PMC5122483

[3] To K, Cao R, Yegiazaryan A, Owens J, Venketaraman V. 2020. General overview of nontuberculous mycobacteria opportunistic pathogens: *Mycobacterium avium* and *Mycobacterium abscessus*. J Clin Med 9:2541. 10.3390/jcm9082541. PMC7463534 32781595

[4] Kwon YS, Daley CL, Koh WJ. 2019. Managing antibiotic resistance in nontuberculous mycobacterial pulmonary disease: challenges and new approaches. Expert Rev Respir Med 13:851–861. 10.1080/17476348.2019.1638765. 31256694

[5] Johansen MD, Herrmann J-L, Kremer L. 2020. Non-tuberculous mycobacteria and the rise of *Mycobacterium abscessus*. Nat Rev Microbiol 18:392–407. 10.1038/s41579-020-0331-1. 32086501

[6] Luthra S, Rominski A, Sander P. 2018. The role of antibiotic-target-modifying and antibiotic-modifying enzymes in *Mycobacterium abscessus* drug resistance. Front Microbiol 9:2179. 10.3389/fmicb.2018.02179. 30258428 PMC6143652

[7] Strnad L, Winthrop KL. 2018. Treatment of *Mycobacterium abscessus* complex. Semin Respir Crit Care Med 39:362–376. 10.1055/s-0038-1651494. 30071551

[8] Daley CL, Iaccarino JM, Lange C, Cambau E, Wallace RJ, Jr, Andrejak C, Böttger EC, Brozek J, Griffith DE, Guglielmetti L, Huitt GA, Knight SL, Leitman P, Marras TK, Olivier KN, Santin M, Stout JE, Tortoli E, van Ingen J, Wagner D, Winthrop KL. 2020. Treatment of nontuberculous mycobacterial pulmonary disease: an official ATS/ERS/ESCMID/IDSA clinical practice guideline. Eur Respir J 56:2000535. 10.1183/13993003.00535-2020. 32636299 PMC8375621

[9] Kwak N, Dalcolmo MP, Daley CL, Eather G, Gayoso R, Hasegawa N, Jhun BW, Koh W-J, Namkoong H, Park J, Thomson R, van Ingen J, Zweijpfenning SMH, Yim J-J. 2019. *Mycobacterium abscessus* pulmonary disease: individual patient data meta-analysis. Eur Respir J 54:1801991. 10.1183/13993003.01991-2018. 30880280

[10] Lopeman RC, Harrison J, Desai M, Cox JAG. 2019. *Mycobacterium abscessus*: environmental bacterium turned clinical nightmare. Microorganisms 7:90. 10.3390/microorganisms7030090. PMC6463083 30909391

[11] Jarand J, Levin A, Zhang L, Huitt G, Mitchell JD, Daley CL. 2011. Clinical and microbiologic outcomes in patients receiving treatment for *Mycobacterium abscessus* pulmonary disease. Clin Infect Dis 52:565–571. 10.1093/cid/ciq237. 21292659

[12] Pasipanodya JG, Ogbonna D, Ferro BE, Magombedze G, Srivastava S, Deshpande D, Gumbo T. 2017. Systematic review and meta-analyses of the effect of chemotherapy on pulmonary *Mycobacterium abscessus* outcomes and disease recurrence. Antimicrob Agents Chemother 61:e01206-17. 10.1128/AAC.01206-17. 28807911 PMC5655093

[13] Fennelly KP, Ojano-Dirain C, Yang Q, Liu L, Lu L, Progulske-Fox A, Wang GP, Antonelli P, Schultz G. 2016. Biofilm formation by *Mycobacterium abscessus* in a lung cavity. Am J Respir Crit Care Med 193:692–693. 10.1164/rccm.201508-1586IM. 26731090

[14] Qvist T, Eickhardt S, Kragh KN, Andersen CB, Iversen M, Hoiby N, Bjarnsholt T. 2015. Chronic pulmonary disease with *Mycobacterium abscessus* complex is a biofilm infection. Eur Respir J 46:1823–1826. 10.1183/13993003.01102-2015. 26493807

[15] Yam Y-K, Alvarez N, Go M-L, Dick T. 2020. Extreme drug tolerance of *Mycobacterium abscessus* “persisters”. Front Microbiol 11:359. 10.3389/fmicb.2020.00359. 32194537 PMC7064438

[16] Daniel-Wayman S, Abate G, Barber DL, Bermudez LE, Coler RN, Cynamon MH, Daley CL, Davidson RM, Dick T, Floto RA, Henkle E, Holland SM, Jackson M, Lee RE, Nuermberger EL, Olivier KN, Ordway DJ, Prevots DR, Sacchettini JC, Salfinger M, Sassetti CM, Sizemore CF, Winthrop KL, Zelazny AM. 2019. Advancing translational science for pulmonary nontuberculous mycobacterial infections. A road map for research. Am J Respir Crit Care Med 199:947–951. 10.1164/rccm.201807-1273PP. 30428263 PMC6467310

[17] Wu ML, Aziz DB, Dartois V, Dick T. 2018. NTM drug discovery: status, gaps and the way forward. Drug Discov Today 23:1502–1519. 10.1016/j.drudis.2018.04.001. 29635026 PMC6078814

[18] Egorova A, Jackson M, Gavrilyuk V, Makarov V. 1 March 2021. Pipeline of anti-*Mycobacterium abscessus* small molecules: repurposable drugs and promising novel chemical entities. Med Res Rev 10.1002/med.21798. PMC8217127 33645845

[19] Richter A, Strauch A, Chao J, Ko M, Av-Gay Y. 2018. Screening of preselected libraries targeting *Mycobacterium abscessus* for drug discovery. Antimicrob Agents Chemother 62:e00828-18. 10.1128/AAC.00828-18. 30012760 PMC6125491

[20] Dupont C, Viljoen A, Dubar F, Blaise M, Bernut A, Pawlik A, Bouchier C, Brosch R, Guérardel Y, Lelièvre J, Ballell L, Herrmann JL, Biot C, Kremer L. 2016. A new piperidinol derivative targeting mycolic acid transport in *Mycobacterium abscessus*. Mol Microbiol 101:515–529. 10.1111/mmi.13406. 27121350

[21] Low JL, Wu M-L, Aziz DB, Laleu B, Dick T. 2017. Screening of TB actives for activity against nontuberculous Mycobacteria delivers high hit rates. Front Microbiol 8:1539. 10.3389/fmicb.2017.01539. 28861054 PMC5559473

[22] Ballell L, Bates RH, Young RJ, Alvarez-Gomez D, Alvarez-Ruiz E, Barroso V, Blanco D, Crespo B, Escribano J, Gonzalez R, Lozano S, Huss S, Santos-Villarejo A, Martin-Plaza JJ, Mendoza A, Rebollo-Lopez MJ, Remuinan-Blanco M, Lavandera JL, Perez-Herran E, Gamo-Benito FJ, Garcia-Bustos JF, Barros D, Castro JP, Cammack N. 2013. Fueling open-source drug discovery: 177 small-molecule leads against tuberculosis. ChemMedChem 8:313–321. 10.1002/cmdc.201200428. 23307663 PMC3743164

[23] Rebollo-Lopez MJ, Lelievre J, Alvarez-Gomez D, Castro-Pichel J, Martinez-Jimenez F, Papadatos G, Kumar V, Colmenarejo G, Mugumbate G, Hurle M, Barroso V, Young RJ, Martinez-Hoyos M, Gonzalez del Rio R, Bates RH, Lopez-Roman EM, Mendoza-Losana A, Brown JR, Alvarez-Ruiz E, Marti-Renom MA, Overington JP, Cammack N, Ballell L, Barros-Aguire D. 2015. Release of 50 new, drug-like compounds and their computational target predictions for open source anti-tubercular drug discovery. PLoS One 10:e0142293. 10.1371/journal.pone.0142293. 26642067 PMC4671658

[24] Das S, Garg T, Srinivas N, Dasgupta A, Chopra S. 2019. Targeting DNA gyrase to combat *Mycobacterium tuberculosis*: an update. Curr Top Med Chem 19:579–593. 10.2174/1568026619666190304130218. 30834837

[25] McKie SJ, Neuman KC, Maxwell A. 2021. DNA topoisomerases: advances in understanding of cellular roles and multi-protein complexes via structure-function analysis. Bioessays 43:2000286. 10.1002/bies.202000286. PMC7614492 33480441

[26] Falzon D, Schunemann HJ, Harausz E, Gonzalez-Angulo L, Lienhardt C, Jaramillo E, Weyer K. 2017. World Health Organization treatment guidelines for drug-resistant tuberculosis, 2016 update. Eur Respir J 49:1602308. 10.1183/13993003.02308-2016. 28331043 PMC5399349

[27] Novosad SA, Beekmann SE, Polgreen PM, Mackey K, Winthrop KL, Team MS, M. abscessus Study Team. 2016. Treatment of *Mycobacterium abscessus* infection. Emerg Infect Dis 22:511–514. 10.3201/eid2203.150828. 26890211 PMC4766900

[28] Choi H, Kim SY, Kim DH, Huh HJ, Ki CS, Lee NY, Lee SH, Shin S, Shin SJ, Daley CL, Koh WJ. 2017. Clinical characteristics and treatment outcomes of patients with acquired macrolide-resistant *Mycobacterium abscessus* lung disease. Antimicrob Agents Chemother 61:e01146-17. 10.1128/AAC.01146-17. 28739795 PMC5610486

[29] Aldred KJ, Blower TR, Kerns RJ, Berger JM, Osheroff N. 2016. Fluoroquinolone interactions with *Mycobacterium tuberculosis* gyrase: enhancing drug activity against wild-type and resistant gyrase. Proc Natl Acad Sci U S A 113:E839–E846. 10.1073/pnas.1525055113. 26792518 PMC4763725

[30] Von Groll A, Martin A, Jureen P, Hoffner S, Vandamme P, Portaels F, Palomino JC, da Silva PA. 2009. Fluoroquinolone resistance in *Mycobacterium tuberculosis* and mutations in gyrA and gyrB. Antimicrob Agents Chemother 53:4498–4500. 10.1128/AAC.00287-09. 19687244 PMC2764174

[31] Farhat MR, Jacobson KR, Franke MF, Kaur D, Sloutsky A, Mitnick CD, Murray M. 2016. Gyrase mutations are associated with variable levels of fluoroquinolone resistance in *Mycobacterium tuberculosis*. J Clin Microbiol 54:727–733. 10.1128/JCM.02775-15. 26763957 PMC4767988

[32] Locher CP, Jones SM, Hanzelka BL, Perola E, Shoen CM, Cynamon MH, Ngwane AH, Wiid IJ, van Helden PD, Betoudji F, Nuermberger EL, Thomson JA. 2015. A novel inhibitor of gyrase B is a potent drug candidate for treatment of tuberculosis and nontuberculosis mycobacterial infections. Antimicrob Agents Chemother 59:1455–1465. 10.1128/AAC.04347-14. 25534737 PMC4325822

[33] Bush NG, Diez-Santos I, Abbott LR, Maxwell A. 2020. Quinolones: mechanism, lethality and their contributions to antibiotic resistance. Molecules 25:5662. 10.3390/molecules25235662. PMC7730664 33271787

[34] Naran K, Moosa A, Barry CE, Boshoff HIM, Mizrahi V, Warner DF. 2016. Bioluminescent reporters for rapid mechanism of action assessment in tuberculosis drug discovery. Antimicrob Agents Chemother 60:6748–6757. 10.1128/AAC.01178-16. 27572410 PMC5075082

[35] Iacobino A, Piccaro G, Pardini M, Fattorini L, Giannoni F. 2021. Moxifloxacin activates the SOS response in *Mycobacterium tuberculosis* in a dose- and time-dependent manner. Microorganisms 9:255. 10.3390/microorganisms9020255. 33513836 PMC7911356

[36] Davies B, Morris T. 1993. Physiological parameters in laboratory animals and humans. Pharm Res 10:1093–1095. 10.1023/a:1018943613122. 8378254

[37] Falzon D, Jaramillo E, Schünemann HJ, Arentz M, Bauer M, Bayona J, Blanc L, Caminero JA, Daley CL, Duncombe C, Fitzpatrick C, Gebhard A, Getahun H, Henkens M, Holtz TH, Keravec J, Keshavjee S, Khan AJ, Kulier R, Leimane V, Lienhardt C, Lu C, Mariandyshev A, Migliori GB, Mirzayev F, Mitnick CD, Nunn P, Nwagboniwe G, Oxlade O, Palmero D, Pavlinac P, Quelapio MI, Raviglione MC, Rich ML, Royce S, Rüsch-Gerdes S, Salakaia A, Sarin R, Sculier D, Varaine F, Vitoria M, Walson JL, Wares F, Weyer K, White RA, Zignol M. 2011. WHO guidelines for the programmatic management of drug-resistant tuberculosis: 2011 update. Eur Respir J 38:516–528. 10.1183/09031936.00073611. 21828024

[38] Pennings LJ, Ruth MM, Wertheim HFL, van Ingen J. 2021. The benzimidazole SPR719 shows promising concentration-dependent activity and synergy against nontuberculous mycobacteria. Antimicrob Agents Chemother 65:e02469-20. 10.1128/AAC.02469-20. 33468478 PMC8097464

[39] Ripoll F, Pasek S, Schenowitz C, Dossat C, Barbe V, Rottman M, Macheras E, Heym B, Herrmann JL, Daffé M, Brosch R, Risler JL, Gaillard JL. 2009. Non mycobacterial virulence genes in the genome of the emerging pathogen *Mycobacterium abscessus*. PLoS One 4:e5660. 10.1371/journal.pone.0005660. 19543527 PMC2694998

[40] Choi G-E, Cho Y-J, Koh W-J, Chun J, Cho S-N, Shin SJ. 2012. Draft genome sequence of *Mycobacterium abscessus* subsp. *bolletii* BD^T^. J Bacteriol 194:2756–2757. 10.1128/JB.00354-12. 22535937 PMC3347169

[41] Cho YJ, Yi H, Chun J, Cho SN, Daley CL, Koh WJ, Shin SJ. 2013. The genome sequence of '*Mycobacterium massiliense*' strain CIP 108297 suggests the independent taxonomic status of the *Mycobacterium abscessus* complex at the subspecies level. PLoS One 8:e81560. 10.1371/journal.pone.0081560. 24312320 PMC3842311

[42] Yee M, Klinzing D, Wei J-R, Gengenbacher M, Rubin EJ, Dick T. 2017. Draft genome sequence of *Mycobacterium abscessus* Bamboo. Genome Announc 5:e00388-17. 10.1128/genomeA.00388-17. 28522728 PMC5477336

[43] Dick T, Shin SJ, Koh WJ, Dartois V, Gengenbacher M. 2019. Rifabutin is active against *Mycobacterium abscessus* in mice. Antimicrob Agents Chemother 64:e01943-19. 10.1128/AAC.01943-19. PMC6985736 31767722

[44] Aziz DB, Low JL, Wu ML, Gengenbacher M, Teo JWP, Dartois V, Dick T. 2017. Rifabutin is active against *Mycobacterium abscessus* complex. Antimicrob Agents Chemother 61:e00155-17. 10.1128/AAC.00155-17. 28396540 PMC5444174

[45] Dartois CGY, Markwell RE, Nadler GMMG, Pearson ND. December 2002. Bicyclic nitrogen-containing heterocyclic derivatives for use as antibacterials. US patent WO 02/096907.

[46] Brookes G, Davies DT, Jones GE, Markwell RE, Pearson ND. October 2003. Nitrogen-containing bicyclic heterocycles for use as antibacterials. US patent WO 03/087098.

[47] Negatu DA, Liu JJJ, Zimmerman M, Kaya F, Dartois V, Aldrich CC, Gengenbacher M, Dick T. 2018. Whole-cell screen of fragment library identifies gut microbiota metabolite indole propionic acid as antitubercular. Antimicrob Agents Chemother 62:e01571-17. 10.1128/AAC.01571-17. 29229639 PMC5826148

[48] Gengenbacher M, Duque-Correa MA, Kaiser P, Schuerer S, Lazar D, Zedler U, Reece ST, Nayyar A, Cole ST, Makarov V, Barry CE, III, Dartois V, Kaufmann SHE. 2017. NOS2-deficient mice with hypoxic necrotizing lung lesions predict outcomes of tuberculosis chemotherapy in humans. Sci Rep 7:8853. 10.1038/s41598-017-09177-2. 28821804 PMC5562869

[49] Yang T, Moreira W, Nyantakyi SA, Chen H, Aziz DB, Go ML, Dick T. 2017. Amphiphilic indole derivatives as antimycobacterial agents: structure-activity relationships and membrane targeting properties. J Med Chem 60:2745–2763. 10.1021/acs.jmedchem.6b01530. 28290692

[50] Negatu DA, Yamada Y, Xi Y, Go ML, Zimmerman M, Ganapathy U, Dartois V, Gengenbacher M, Dick T. 2019. Gut microbiota metabolite indole propionic acid targets tryptophan biosynthesis in *Mycobacterium tuberculosis*. mBio 10:e02781-18. 10.1128/mBio.02781-18. 30914514 PMC6437058

[51] Stover CK, de la Cruz VF, Fuerst TR, Burlein JE, Benson LA, Bennett LT, Bansal GP, Young JF, Lee MH, Hatfull GF, Snapper SB, Barletta RG, Jacobs WR, Bloom BR. 1991. New use of BCG for recombinant vaccines. Nature 351:456–460. 10.1038/351456a0. 1904554

[52] Goude R, Parish T. 2009. Electroporation of mycobacteria. Methods Mol Biol 465:203–215. 10.1007/978-1-59745-207-6_13. 20560076

[53] Blower TR, Williamson BH, Kerns RJ, Berger JM. 2016. Crystal structure and stability of gyrase-fluoroquinolone cleaved complexes from *Mycobacterium tuberculosis*. Proc Natl Acad Sci U S A 113:1706–1713. 10.1073/pnas.1525047113. 26792525 PMC4763791

[54] Andreu N, Zelmer A, Fletcher T, Elkington PT, Ward TH, Ripoll J, Parish T, Bancroft GJ, Schaible U, Robertson BD, Wiles S. 2010. Optimisation of bioluminescent reporters for use with mycobacteria. PLoS One 5:e10777. 10.1371/journal.pone.0010777. 20520722 PMC2875389

[55] Nash KA, Brown-Elliott BA, Wallace RJ. 2009. A novel gene, erm(41), confers inducible macrolide resistance to clinical isolates of *Mycobacterium abscessus* but is absent from *Mycobacterium chelonae*. Antimicrob Agents Chemother 53:1367–1376. 10.1128/AAC.01275-08. 19171799 PMC2663066

