# Supplementary material for: Piperidine-4-Carboxamides Target DNA Gyrase in Mycobacterium abscessus
Source: Antimicrob Agents Chemother. 2021 Jul 16;65(8):e00676-21. doi: 10.1128/AAC.00676-21 (PMC8284461; doi:10.1128/AAC.00676-21)
Supplement: Supplemental file 1 — Supplemental Figures S1 to S3. Download AAC00676-21_Supp_1_seq9.pdf, PDF file, 0.5 MB [file aac00676-21_supp_1_seq9.pdf]

Supplementary Information

Piperidine-4-carboxamides target DNA gyrase in *Mycobacterium abscessus*

Dereje Abate Negatu, Andreas Beuchel, Abdeldjalil Madani, Nadine Alvarez, Chao Chen, Wassihun Wedajo Aragaw, Matthew D. Zimmerman, Benoît Laleu, Martin Gengenbacher, Véronique Dartois, Peter Imming, Thomas Dick

Supplementary Figures

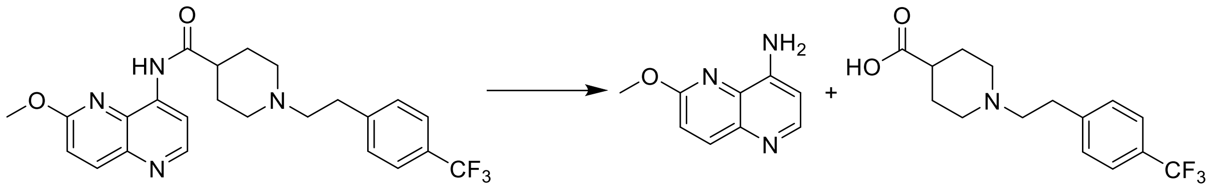

**844-TFM**	**Product 1**	**Product 2**
ESI-FTMS *m/z* $[M+H]^+$ calc. for $C_{24}H_{26}F_3N_4O_2$ 459.2008, found 459.2002	ESI-FTMS *m/z* $[M+H]^+$ calc. for $C_9H_{10}N_3O$ 176.0824, found 176.0818	ESI-FTMS *m/z* $[M+H]^+$ calc. for $C_{15}H_{19}F_3NO_2$ 302.1368, found 302.1362

FIG S1. Identification of two major metabolites generated in mouse plasma. ESI-FTMS, Electrospray ionization - Fourier Transform Mass Spectrometer. Q-Exactive high resolution extracted ion chromatograms of the cleavage products of 844-TFM are shown in Fig. S2.

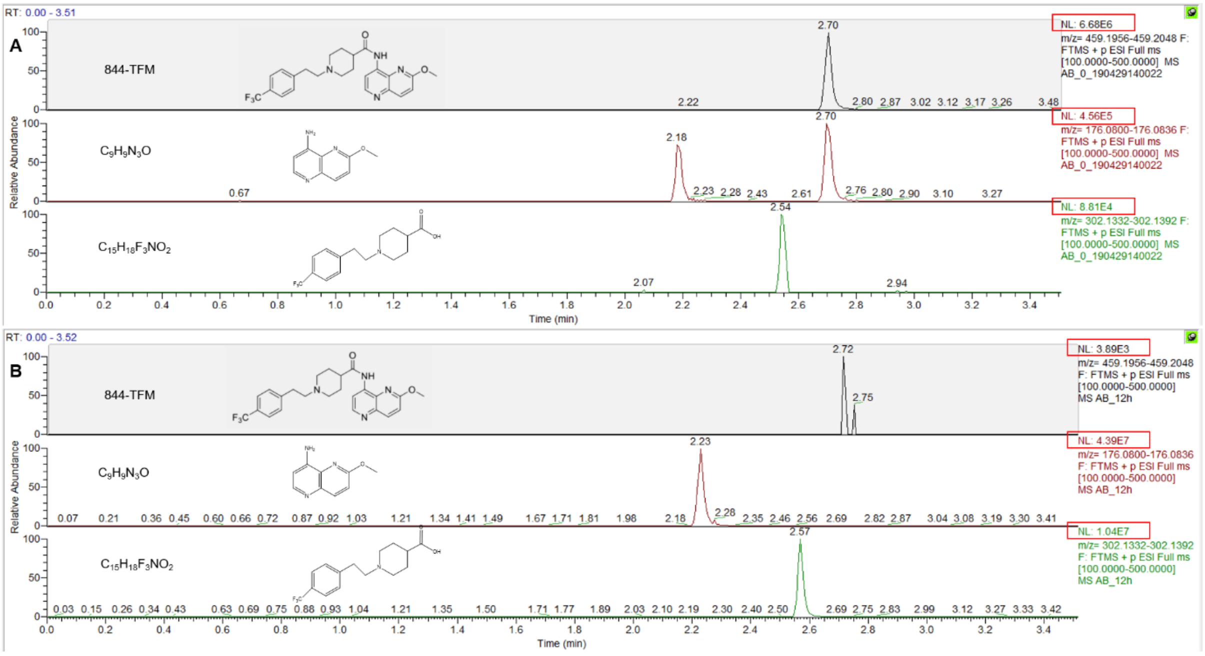

FIG S2. Extracted ion chromatogram of 844-TFM and its two metabolites $C_9H_9N_3O$ and $C_{15}H_{18}F_3NO_2$ in mouse plasma. 844-TFM was added to mouse plasma and samples were analyzed immediately after 30 s (A) or after incubation at 37℃ for 12 h (B). (A) shows a high signal intensity for 844-TFM whereas its two metabolites $C_9H_9N_3O$ and $C_{15}H_{18}F_3NO_2$ show a lower signal intensity compared to the original substance. (B) The intensities of the two 844-TFM-metabolites exceed that of the parent molecule. NL in red boxes, Normalized Level, i.e. signal intensity. Mass accuracy 5 ppm. The results show hydrolysis of 844-TFM's amide bond in mouse plasma.

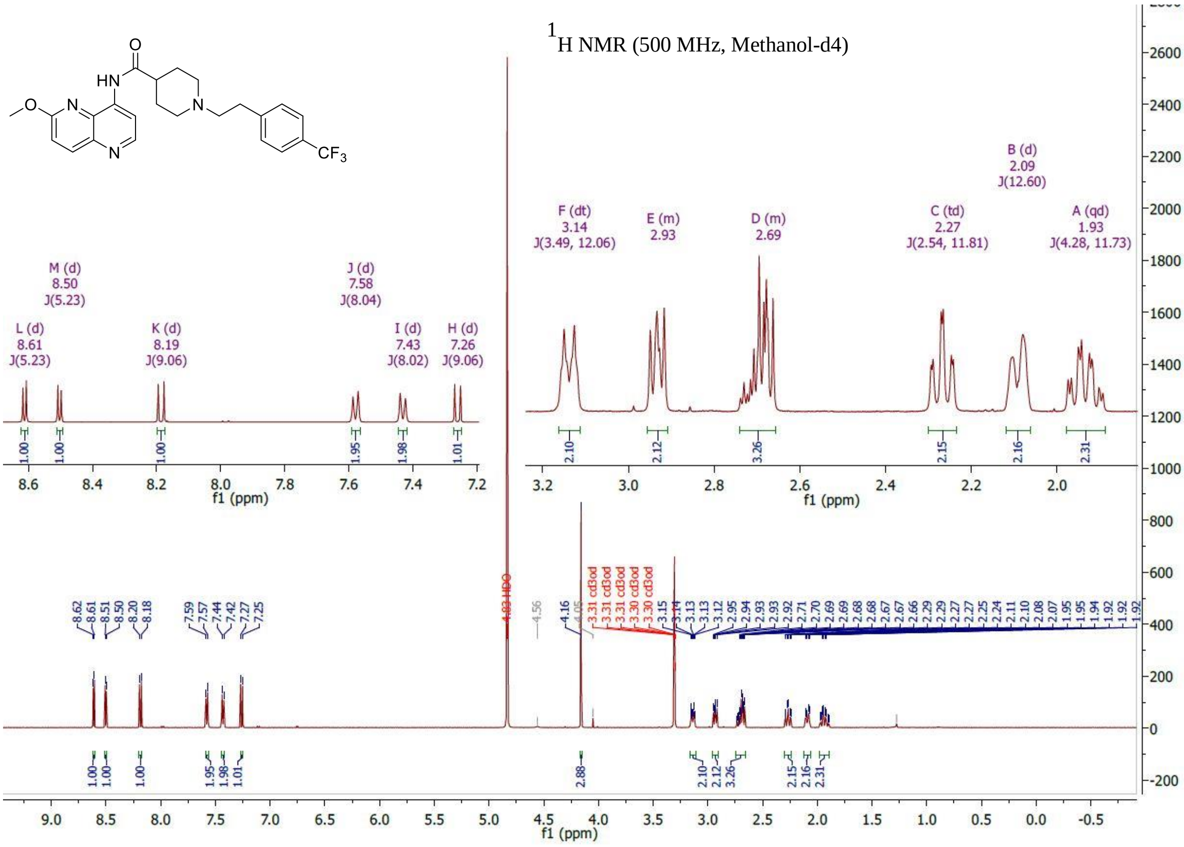

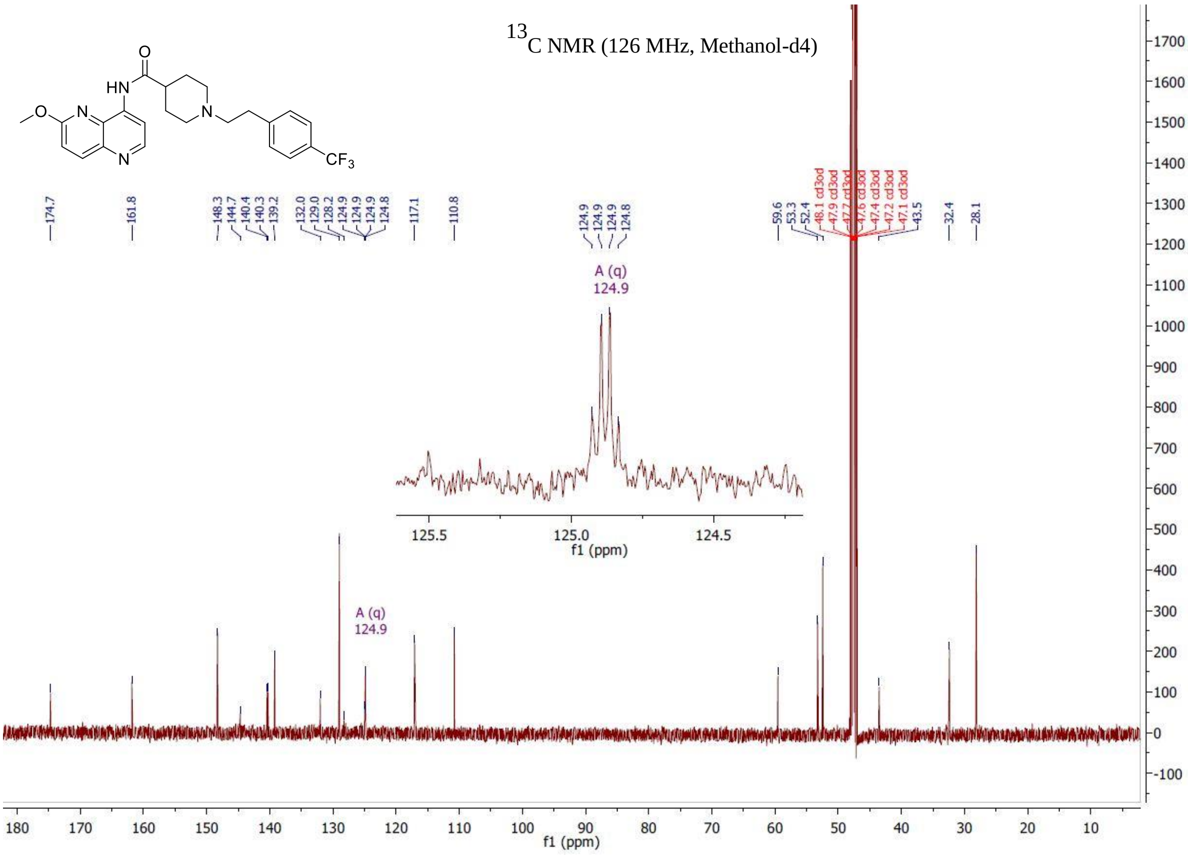

FIG S3. ^{1}H and ^{13}C NMR spectra of 844-TFM
